# *In silico *and biological survey of transcription-associated proteins implicated in the transcriptional machinery during the erythrocytic development of *Plasmodium falciparum*

**DOI:** 10.1186/1471-2164-11-34

**Published:** 2010-01-15

**Authors:** Emmanuel Bischoff, Catherine Vaquero

**Affiliations:** 1 Institut Pasteur, Unité d'Immunologie Moléculaire des Parasites, CNRS URA 2581, 25-28 rue du Dr Roux, 75724, Paris cedex 15, France; 2 Institut Pasteur, Unité de Génétique et Génomique des Insectes Vecteurs, CNRS URA 3012, 25-28 rue du Dr Roux, 75724, Paris cedex 15, France; 3 UMRS 945, Inserm & Université Pierre et Marie Curie (UPMC Paris 6), 91 Bd de l'Hôpital, 75013 Paris, France

## Abstract

**Background:**

Malaria is the most important parasitic disease in the world with approximately two million people dying every year, mostly due to *Plasmodium falciparum *infection. During its complex life cycle in the Anopheles vector and human host, the parasite requires the coordinated and modulated expression of diverse sets of genes involved in epigenetic, transcriptional and post-transcriptional regulation. However, despite the availability of the complete sequence of the *Plasmodium falciparum *genome, we are still quite ignorant about *Plasmodium *mechanisms of transcriptional gene regulation. This is due to the poor prediction of nuclear proteins, cognate DNA motifs and structures involved in transcription.

**Results:**

A comprehensive directory of proteins reported to be potentially involved in *Plasmodium *transcriptional machinery was built from all *in silico *reports and databanks. The transcription-associated proteins were clustered in three main sets of factors: general transcription factors, chromatin-related proteins (structuring, remodelling and histone modifying enzymes), and specific transcription factors. Only a few of these factors have been molecularly analysed. Furthermore, from transcriptome and proteome data we modelled expression patterns of transcripts and corresponding proteins during the intra-erythrocytic cycle. Finally, an interactome of these proteins based either on *in silico *or on 2-yeast-hybrid experimental approaches is discussed.

**Conclusion:**

This is the first attempt to build a comprehensive directory of potential transcription-associated proteins in *Plasmodium*. In addition, all complete transcriptome, proteome and interactome raw data were re-analysed, compared and discussed for a better comprehension of the complex biological processes of *Plasmodium falciparum *transcriptional regulation during the erythrocytic development.

## Background

The complete genome sequencing of *Plasmodium *[1] allowed the prediction of around 5,500 Open Reading Frames (ORFs). Regulation of gene expression of this unicellular eukaryote is probably governed, as for all eukaryotes, by common mechanisms including epigenetic, transcriptional as well as post-transcriptional regulation. During the pathogenic intra-erythrocytic development, the parasite undergoes a highly complex life cycle characterized by a succession of very different developmental stages. However, the molecular events controlling stage development are not known. During the last decade, investigations of several *Plasmodium *transcripts [2-5] were followed by comprehensive analyses of transcript levels *via *pan-genomic micro-array approaches [6,7]. All these data revealed that the *Plasmodium *genome is transcriptionally active, since at least 60% of the *Plasmodium *transcripts were differentially and tightly regulated throughout the developmental transition. Indeed, the morphological changes observed throughout the erythrocytic cycle (parasite multiplication from ring to schizont and reinvasion of host cells with occasional arrest of proliferation and differentiation into gametocytes) are probably governed by the fine tuning of coordinate expression of genes. One can reasonably assume that gene expression is under the control of various transcriptional mechanisms: structuring and remodelling of chromatin, combinatorial interactions between *trans *and *cis *regulatory elements (RE) even though post-transcriptional regulation probably participates significantly to gene regulation as well (for review [8-11]).

Despite a growing number of computational and molecular experimental approaches developed to understand gene regulation in *Plasmodium*, knowledge of transcriptional and post-transcriptional regulation is clearly quite limited and the annotation of the *cis *and *trans *elements implicated in gene transcription is poor. In addition, the relative contributions of the mechanisms governing gene regulation (transcription versus post-transcription) are still a subject of debate, awaiting a number of additional functional experiments. Indeed, only a small number of putative specific Transcription Factors (TFs) could be identified when compared to other unicellular eukaryotic organisms [12]. If a small number of specific transcription factors is a real characteristic of *Plasmodium*, this might in turn account for the unusual mechanisms of gene regulation involving a major contribution of epigenetic and post-transcriptional machineries [10]. Conversely, around 60% of orphan genes among the 5,500 predicted ORFs might lead to the apparent dearth of potential Transcription-Associated Proteins (TAPs) in *Plasmodium*. The high A/T rich genome probably contributes to this poor annotation due to a weak similarity of *Plasmodium *factors with the amino acid sequences of eukaryotic proteins listed in DataBanks including TRANSFAC^® ^the Transcription Factor Database [13].

Nevertheless, *Plasmodium *shares with other eukaryotes common mechanisms involved in gene transcriptional regulation. This assumption is supported by the nucleosome organization, structure of promoters and monocistronic mRNA transcription as well as by the RNA messenger organization: occurrence of introns, even if short and scarce, standard splice and acceptor sites, and conventional 5' and 3' untranslated regions that are capped and polyadenylated, respectively. Indeed, several genes of *Plasmodium *have been investigated and appeared to share the overall features of eukaryotic genes indicating that *Plasmodium *gene regulation might resemble that of eukaryotes, even though the transcriptional regulatory elements might be more divergent [8,14]. Since the parasite's development is characterized by coordinate expression of genes, it is realistic to consider that, as for all eukaryotes, the modulation of transcription efficiency in addition to epigenetic regulation should be under the control of combinatorial interactions between specific transcription factors with their cognate *cis *RE present within the promoters. In addition, the level of transcription might be modulated by the diversity and the hierarchy of affinity of different members of a family of transcription factors towards a variety of DNA motifs present in the promoters. The transcriptional control would also depend on the availability of the factors throughout the parasite cycle. Very few interactions between such factors and DNA motifs have been investigated in *Plasmodium *either with factor identification [5,15-17] or without [18,19] and the best studied interactions concern *var *gene regulation (for review [20]).

Despite the enormous effort of the Wellcome Trust Sanger Institute GeneDB and the PlasmoDB teams to improve the genome annotation of *P. falciparum*, TAP prediction still remains elusive. Genome mining and proteome analysis highlighted the apparent small number of recognizable specific TFs [21]. This led the *Plasmodium *community to favour a paucity of specific TFs conferring a higher control of gene regulation at epigenetic and post-transcriptional levels when compared to other eukaryotes. However, it cannot be ruled out that this apparent lack of specific TFs resides in the complexity of annotation and much work remains to be done before reaching any clear-cut conclusion. Indeed, *in silico *prediction using successively two computational procedures [21,22] led to the annotation of most of the general TFs implicated in the basal transcriptional machinery. Furthermore, in addition to the four histone proteins [23] composing nucleosomes and the modifying enzymes implicated in the histone code, a rather high number of chromatin-structuring and remodelling factors, was predicted in *Plasmodium*. As regards the TRANSFAC classification [24] a number of factors belonging to two super-classes: zinc-coordinating DNA-binding domains [21] and ApiAP2 proteins [25] have been *in silico *annotated in the parasite. Conversely, a lack of hits for conserved DNA binding domains including homeodomains, MADS, bZip, bHLH and GATA fingers, and FKH domains was observed in the genome [26]. Only a few factors have been analysed for their interaction with their cognate RE. Despite the observation that some transcription factors can bind to specific DNA domains present within a number of promoters [8,27,28], some remained uncharacterized [18]. However, some specific TAPs were analysed at the molecular level. Several years ago, after annotation by sequence homology of different sets of potential TAPs, our functional studies undertaken with PfMyb1 (PF13_0088) a member of the HTH sub-family with tryptophan domains demonstrated for the first time that this factor was capable to interact specifically with DNA motifs present in *Plasmodium *promoters as also observed for PfMyb2 (PF10_0327) (Coetzer and Vaquero, unpublished results). In addition, PfMyb1 was capable to modulate expression of several targeted genes [5,15]. Recently, a functional study of three members of the ApiAP2 family was reported [16,17]. The next challenge will be to determine novel *Plasmodium *specific TAPs that have never been found in other eukaryotes.

Herein, we have used *in silico *predictions [12,25,26] and our contribution focused on the factors involved in global transcriptional regulation of messenger RNA in order to propose a comprehensive directory of TAPs grouped in four classes and sub-classes. The specific transcription factors were clustered according to TRANSFAC nomenclature. Also, we modelled their transcriptome and proteome expression profiles throughout erythrocytic development and protein-protein interaction network from reports described in the literature [6,7,29-32]. The results obtained on a small number of previously annotated specific factors were also taken into account. This study was carried out to unravel, at least to some extent, the nuclear transcription network occurring during the life-cycle transitions of the erythrocytic *Plasmodium *cycle.

## Results and discussion

Since a number of reports showed that the transcriptome profiles change [6,7] during the parasite erythrocytic development, it was reasonable to assume that different sets of TAPs were essential to govern the fine tuning of transcriptional regulation during the erythrocytic cycle. However, only a few *in silico *studies concerning *P. falciparum *TAPs have been published. Coulson and collaborators [12] used two strategies to identify the TFs of *P. falciparum*: 51 HMM profiles from TRANSFAC and a sequence similarity clustering method were used to identify 156 TAP including specific transcription factors in the *P. falciparum *genome. However, among these 156 TAPs only 129 were implicated in transcription of messenger RNA encoded by the nuclear genome. Callebaut and colleagues [22] used a two dimensional hydrophobic cluster analysis (HCA) to identify 10 additional general TFs. Finally, Balaji *et al*. [25] reported numerous members of the ApiAP2 family of specific transcription factors.

It is important to define what we have considered as TAPs listed in the directory (Table 1). It comprises a global classification of all annotated TAPs appearing in the literature relative to messenger RNA transcription. All factors associated with transcription of ribosomal and transfer RNAs and those associated with apicoplast, mitochondria and mini-chromosome maintenance were excluded from the list.

**Table 1:** Directory of the *Plasmodium *transcription-associated proteins: number and functions.

I. General Transcription Factors (GTF)	
1. Polymerase core subunits	12
2. Polymerase accessory proteins	2
3. General coactivators involved in initiation	25
4. General coactivators involved in initiation and elongation	13
5. General coactivators involved elongation	4
*sub-total*	56
II. Chromatin-related Transcription Factors (CTF)	
1. Histones	8
2. Chromatin structuring factors	18
3. Chromatin remodelling factors	13
4. Modifying enzymes	24
*sub-total*	63

III. Specific Trancription Factors (STF)	
0 SC. Apicomplexan specifc AP2	27
2 SC. Zinc finger	37
3 SC. Helix Turn Helix	8
4 SC. beta-Scaffold Factors with Minor Groove Contacts	1
*sub-total*	73

IV. TAP partners	10

*Total*	202

The number of all sets of transcription-associated proteins are listed according to their classes and sub-classes named with respect to their potential functional implication in transcription of the messenger RNA.

### I. Comprehensive directory of factors associated with *Plasmodium *transcription

Combining the aforementioned data and the Pfam database annotations, we selected 109 HMM profiles to scan the whole *Plasmodium *genome in order to identify the general transcription factors: the RNA polymerases and factors composing the Pre-Initiation Complex (PIC), all factors implicated in chromatin remodelling and enzymatic modifications as well as the specific transcription factors. It is of note that Coulson and colleagues used only 51 HMM followed by a sequence similarity clustering method (see for details Methods). Our functional annotation was assessed by two strategies: 1) by comparing the domain organization of each protein to annotated proteins sharing the same domain organization in the Pfam database and 2) by searching orthologs in model organisms. This double check procedure minimized false positive TAPs (but increased false negatives). When the TAP candidates were annotated as belonging to a protein complex in model organisms, all *P. falciparum *orthologs of the complex were searched and subunits that were missed using the HMM strategy were added to the final list of TAPs. In addition, it was possible to identify, based mainly on chromatin binding domains and protein-protein interactions, in the *Plasmodium *3D7 genomic sequence completed in 2002 [1], diverse protein domains associated with functional features of eukaryotic TAPs in charge of transcription regulation and in turn involved in a variety of cellular processes. This directory was used to link each putative factor with its expected function in order to increase the knowledge of *Plasmodium *transcriptional machinery. Our *in silico *procedure increased the number of HMM and thus the quality of functional annotation and we identified 202 TAPs. Among these factors are the previously annotated factors: 104 by Coulson *et al*., 20 by Callebaut *et al*., 27 ApiAP2 by Balaji *et al*., 27 recovered from PlasmoDB and 34 novel annotations including several previously identified by Vaquero's team. They were classified into four functional classes and sub-classes. For specific TFs, we used the TRANSFAC database classification.

The four classes are: I) the general transcription factors (GTFs) with all polymerase subunits and general co-activators involved in initiation and elongation of mRNA transcripts; II) the factors involved in chromatin-structuring and remodelling of the nucleosomes as well as the histones implicated in the nucleosome structure. In this section all modifying enzymes associated with the epigenetic code governing histone modifications and therefore accountable for the modulation of transcription level were added. For simplification, all these factors were included in chromatin-related transcription factors (CTFs); III) all specific transcription factors (STFs) annotated so far, including the recent group of ApiAP2, whose interactions with specific DNA sequences (binding motifs) participate in transcription efficiency. The STFs have been clustered according to TRANSFAC classification. Finally, we listed a few members of TAP partners in class IV. A brief description of all TAP is summarized in Table 1, listed in Tables 2, 3, 4 and 5 and detailed in additional file 1.

**Table 2:** List of *P. falciparum *General Transcription Factors sorted by functions.

Sub-classes	Gene ID	Annotation
1. Polymerase core subunit	MAL13P1.213	RNA POLYMERASE I/II/III::Rbp-12
	PF07_0027	RNA POLYMERASE I/II/III::Rpb-10
	PF13_0341	RNA POLYMERASE I/II/III::Rpb-5
	PFC0155c	RNA POLYMERASE I/II/III::Rpb-6
	PFL0665c	RNA POLYMERASE I/II/III::Rpb-8
	PFC0805w	RNA POLYMERASE II::Rpb-1
	PF13_0023	RNA POLYMERASE II::Rpb-11
	PFB0715w	RNA POLYMERASE II::Rpb-2
	PFI1130c	RNA POLYMERASE II::Rpb-3
	PFB0245c	RNA POLYMERASE II::Rpb-4
	PF10_0269	RNA POLYMERASE II::Rpb-7
	PFA0505c	RNA POLYMERASE II::Rpb-9
2. Polymerase accessories	PF08_0037	RNA polymerase II mediator complex protein MED7
	PF14_0718	RNA polymerase II-associated factor SOH1
3. General coactivators involved in initiation	PF13_0043	CCAAT Box-binding complex::CBF-C/NF-Y-A
	PF11_0477	CCAAT Box-binding complex::CBF-C/NF-Y-B
	PF14_0374	CCAAT Box-binding complex::CBF-C/NF-Y-C
	MAL7P1.78	TFIIA::TFIIA alpha
	PFI1630c	TFIIA::TFIIA gamma
	PFL2435w	TFIIA::TFIIA gamma
	PFA0525w	TFIIB::TFIIB
	PFE0415w	TFIIB::TFIIB-like
	PFL1645w	TFIID::TAF1
	MAL7P1.134	TFIID::TAF2
	PFI1425w	TFIID::TAF7
	PFE0305w	TFIID::TBP
	PF14_0267	TFIID::TBP-like
	PFE1110w	TFIID/SAGA::TAF10
	MAL7P1.86	TFIIE::TFIIE alpha
	MAL13P1.360	TFIIE::TFIIE beta
	PFC1055w	TFIIH core p62/TFB1
	PF14_0398	TFIIH core TFB5
	PF13_0022	TFIIH::cyclin K
	PFE0610c	TFIIH::MAT1
	PF13_0279	TFIIH::p34
	MAL13P1.76	TFIIH::p44
	PFL2125c	TFIIH::p52
	PF10_0369	TFIIH::XPB
	PFI1650w	TFIIH::XPD
4. General coactivators involved in initiation and elongation	PF11_0458	TFIIF::TFIIF beta
	MAL8P1.104	CCR4-NOT complex::CAF1
	PFE0375w	CCR4-NOT complex::CAF40
	PFA0350w	CCR4-NOT complex::CCR4
	PFC0850c	CCR4-NOT complex::CCR4
	PFE0980c	CCR4-NOT complex::CCR4
	PF11_0049	CCR4-NOT complex::NOT1
	PF14_0170	CCR4-NOT complex::NOT1
	PF11_0297	CCR4-NOT complex::NOT2
	PF10_0062	CCR4-NOT complex::NOT3/NOT5
	PFL1705w	CCR4-NOT complex::NOT4
	PF14_0241	General transcription factor BTF3
	PF11_0293	Transcriptional co-activator MBF1
5. General coactivators involved elongation	PF10_0293	DSIF::SPT4
	PFF0535c	DSIF::SPT5
	PF14_0059	SPT6
	PF07_0057	TFIIS

**Table 3:** List of *P. falciparum *Chromatin-related Factors sorted by functions.

Sub-classes	Gene ID	Annotation
1. Histones	PFC0920w	Histone H2A
	PFF0860c	Histone H2A
	PF07_0054	Histone H2B
	PF11_0062	Histone H2B
	PF13_0185	Histone H3
	PFF0510w	histone H3
	PFF0865w	Histone H3
	PF11_0061	Histone H4
2. Chromatin structuration	PF10_0328	bromodomain protein
	PF14_0724	bromodomain protein
	PFA0510w	bromodomain protein
	PFL0635c	bromodomain protein
	PF11_0418	chromodomain protein
	PFL1005c	PfHP1 (chromodomain protein)
	PFB0875c	Chromatin-binding protein, putative
	PFF1185w	ISW1::ISW1 homologue
	PF10_0232	SAGA/SILK::CHD1 homologue
	PFB0730w	SWI/SNF::SNF2 homologue
	PF13_0308	SWI/SNF::SNF2 like
	PF08_0048	SWR1::SWR1
	PFF0225w	DNA helicase
	MAL8P1.65	FUN30 homologue
	PF11_0053	PfSNF2L: chromatin accessibility complex::ISW2 homologue
	PFI0590c	zf-HIT protein
	PF14_0314	chromatin assembly factor 1 p55 subunit, putative
	PFA0520c	chromatin assembly factor 1 protein WD40 domain, putative
3. Chromatin remodelling	PFE0090w	chromosome assembly factor 1, CAF-1
	PF14_0393	FACT::POB3
	PFE0870w	FACT::SPT16
	PF08_0100	SWR1::RVB1 homologue
	PF11_0071	SWR1::RVB1 homologue
	PF13_0330	SWR1::RVB2 homologue
	PF14_0608	SWR1::SWC2 homologue
	PFF1385c	SWR1/NuA4::SWC4 homologue
	PFI0930c	nucleosome assembly protein (PfB7)
	PFL0185c	nucleosome assembly protein 1
	PFL0145c	PfHMGB1
	MAL8P1.72	PfHMGB2
	MAL13P1.290	PfHMGB4
4. Modifying enzymes	MAL13P1.255	adenine-specific methylase
	PF11_0192	HAT, NuA4::ESA1
	PF08_0034	HAT, ADA/SAGA::GCN5
	MAL8P1.200	HAT, histone acetyl transferase
	PF10_0036	HAT, histone acetyl transferase
	PF13_0131	HAT, histone acetyl transferase
	PF14_0350	HAT, histone acetyl transferase
	PFA0465c	HAT, histone acetyl transferase
	PFF1405c	HAT, histone acetyl transferase
	PF10_0078	HDAC, Histone deacetylase
	PF14_0690	HDAC, Histone deacetylase
	PFI1260c	HDAC, Histone deacetylase
	PF13_0152	HDAC, SIR2 family
	PF14_0489	HDAC, SIR2 family
	MAL8P1.111	histone demethylase JHD2
	MAL13P1.19	SAGA/NuA4::TRA1
	MAL13P1.122	SET domain protein
	PF08_0012	SET domain protein
	PF11_0160	SET domain protein
	PFD0190w	SET domain protein
	PFF1440w	SET domain protein
	PFL0690c	SET histone-lysine N-methyltransferase, putative
	MAL7P1.37	SIN3-repressing complex::p18
	MAL8P1.131	SWR1/NuA4::YAF9 homologue

**Table 4:** List of *P. falciparum *Specific Trancription Factors sorted by functions.

Sub-classes	Gene ID	Annotation
0 SC. Apicomplexan specifc AP2	PF13_0026	ApiAP2 developmental TF
	PF14_0271	ApiAP2 developmental TF
	PFF0550w	ApiAP2 developmental TF
	MAL8P1.153	ApiAP2 developmental TF (Early Schizont)
	PF11_0163	ApiAP2 developmental TF (Early Schizont)
	PF14_0533	ApiAP2 developmental TF (Early Schizont)
	PFE0840c	ApiAP2 developmental TF (Early Schizont)
	PFF0200c	ApiAP2 developmental TF (Early Schizont)
	PFF0670w	ApiAP2 developmental TF (Early Schizont)
	PF13_0097	ApiAP2 developmental TF (multiple stages)
	PFD0200c	ApiAP2 developmental TF (multiple stages)
	PF07_0126	ApiAP2 developmental TF (Ring)
	PF14_0079	ApiAP2 developmental TF (Ring)
	PF14_0633	ApiAP2 developmental TF (Ring)
	PFF1100c	ApiAP2 developmental TF (Ring)
	PF11_0091	ApiAP2 developmental TF (Schizont)
	PF11_0404	ApiAP2 developmental TF (Schizont)
	PF11_0442	ApiAP2 developmental TF (Schizont)
	PF13_0235	ApiAP2 developmental TF (Schizont)
	PFD0985w	ApiAP2 developmental TF (Schizont)
	PFL1085w	ApiAP2 developmental TF (Schizont)
	PF10_0075	ApiAP2 developmental TF (Trophozoite)
	PF13_0267	ApiAP2 developmental TF (Trophozoite)
	PF14_0471	ApiAP2 developmental TF (Trophozoite)
	PFI1665w	ApiAP2 developmental TF (Trophozoite)
	PFL1075w	ApiAP2 developmental TF (Trophozoite)
	PFL1900w	ApiAP2 developmental TF (Trophozoite)
2 SC. Zinc finger	MAL13P1.37	Bbox protein
	PF14_0383	Bbox protein
	PFC0345w	Bbox protein
	PFE0895c	Bbox protein
	PF07_0124	MYND finger domain protein
	PFF0105w	MYND finger domain protein
	PFF0350w	MYND finger domain protein
	PF10_0091	zf-C2H2 protein
	PF14_0479	zf-C2H2 protein
	PF14_0559	zf-C2H2 protein
	PF14_0612	zf-C2H2 protein
	PF14_0657	zf-C2H2 protein
	PF14_0707	zf-C2H2 protein
	PFC0690c	zf-C2H2 protein
	PFD0375w	zf-C2H2 protein
	PFD0485w	zf-C2H2 protein
	PFL0455c	zf-C2H2 protein
	PFL0465c	zf-C2H2 protein
	PFL2075c	zf-C2H2 protein
	PFI0470w	zf-C3HC4 protein
	PFL0275w	zf-C3HC4 protein
	MAL8P1.70	zf-CCCH protein
	PF10_0083	zf-CCCH protein
	PF10_0186	zf-CCCH protein
	PF11_0357	zf-CCCH protein
	PF13_0314	zf-CCCH protein
	PF14_0236	zf-CCCH protein
	PF14_0416	zf-CCCH protein
	PF14_0610	zf-CCCH protein
	PF14_0652	zf-CCCH protein
	PFE1145w	zf-CCCH protein
	PFE1245w	zf-CCCH protein
	PFF0095c	zf-CCCH protein
	PFI0325c	zf-CCCH protein
	PFI1335w	zf-CCCH protein
	PFL0510c	zf-CCCH protein
	PF11_0200	U2 snRNP auxiliary factor, small subunit, putative
3 SC. Helix Turn Helix	PF10_0143	ADA/SAGA/SILK::ADA2
	PFL1215c	SWIR
	PFL0290w	Chromatin binding, PfHMGB3
	PF11_0241	DNA binding
	PF13_0088	PfMyb1
	PF10_0327	PfMyb2: cdc5
	PFL0815w	zuotin-related factor 1 (m-phase phosphoprotein 11)
	PF13_0054	transcription factor, putative
4 SC. beta-Scaffold Factors with Minor Groove Contacts	PFA0470c	Cold-shock protein

**Table 5:** List of *P. falciparum *TAP partners sorted by functions.

Gene ID	Annotation
PF08_0129	Calcineurine::protein phosphatase 3 catalytic subunit
PF14_0492	Calcineurine::protein phosphatase 3 regulatory subunit
PFD1095w	BSD-domain protein
PFI0730w	BSD-domain protein
PFI1255w	homologue to Yippee like MOH1
PF10_0079	PHD domain protein
PF11_0429	PHD domain protein
PFL1010c	PHD domain protein
PFL1905w	PHD domain protein
PFF0760w	Transcriptional co-activator ALY

#### I. General transcription factors

The 56 general TFs (except two) presented in class I (Table 2) came from two *in silico *studies performed by Coulson [12] and Callebaut [22]. The first study, based on 51 HMM profiles and a sequence similarity clustering method allowed the annotation of 12 RNA polymerase subunits, two accessory proteins (with the exception of one accessory protein annotated by PlasmoDB), 16 general co-activators and 13 TFII subunits all potentially involved in the initiation of messenger RNA transcription composing the pre-initiation complex (PIC). As previously mentioned, only 13 of the TFII core complex members were identified in the genome, leading to the hypothesis that there are major differences in the transcriptional machinery of *Plasmodium *when compared to other eukaryotes. Nevertheless, nine additional subunits involved in transcription initiation and one TFIIF involved in initiation and elongation were annotated by the second study using a program based on HCA and secondary protein structure, and profile-based search methods (PSI-BLAST) [22]. Therefore, the subunit number of the PIC rose to 39 putative proteins. In addition, we also integrated in this general transcription factor set, 10 CCR4-NOT (nine reported by Coulson and one from our prediction) additional factors involved in initiation and elongation of transcription as well as four co-activators involved in elongation.

#### II. Chromatin-related transcription factors

We included in class II all putative TAPs with domain features that make them good candidates for participating in nucleosomal organization, chromatin remodelling and epigenetic modification. All these 63 proteins, that play a role in epigenetic regulation of transcription to different degrees, were clustered in four different sub-classes (1 to 4, Table 1 and 3).

In sub-class 1, eight histone genes identified in the parasite were listed in PlasmoDB: two H2A, two H2B, three H3 and one H4 [23,33]. Two sets of these genes were bi-directionally localized in the genome with respect to their common putative promoters (PFF0865w-PFF0860c and PF11_0061-PF11_0062 named histone H3-H2A and H4-H2B, respectively). These proteins might be involved in nucleosome architecture at a given time of erythrocytic development. Note that histone H1, interacting with the DNA linker between two nucleosomes, has not yet been found in the *P. falciparum *genome, despite searches with a variety of different computational approaches.

In sub-class 2 were also listed 18 chromatin structuring proteins comprising factors with a) a bromo domain (four putative proteins) that may play a role in assembly or activity of multi-component complexes involved in transcriptional activation; b) a Chromo domain for CHRromatin Organisation MOdifier (two) and c) a SNF2 domain (eight) found in proteins involved in a variety of processes including transcription regulation (e.g., SNF2, STH1, brahma, MOT1). In this sub-class, a factor PfHP1 (PFL1005c) with a chromo domain was very recently reported to be implicated in heterochromatin formation and antigenic variation [34].

Sub-class 3 comprises 13 factors implicated in chromatin remodelling including nucleosome assembly proteins (NAP, PFI0930c and PFL0185c) [35] and four high mobility group box B (HMGB) proteins including PfHMGB1 (PFL0145c) and PfHMGB2 (MAL8P1.72) that were studied molecularly [36,37]. These architectural functions participate in chromatin remodelling and in turn contribute to regulation of gene transcription.

Finally, sub-class 4 contains a large set of 24 factors that take into account all putative enzymes linked to the epigenetic histone code and predicted to modify the efficiency of transcription, mostly Histone Acetyl Transferases (HATs) and Histone DeACetyl transferases (HDACs) including SIR2 proteins. In addition, some methyl transferases (SETs) were also listed as well as one demethylase (MAL8P1.111). Indeed, all these factors govern the transcriptional efficacy either positively or negatively [20,38-40].

#### III. Specific transcription factors, (73)

The specific transcription factors (class III, Table 4) were clustered in four sub-classes corresponding to TRANSFAC classification (0, 2, 3 and 4 super-classes) with a large variation in the number of putative factors from one in super-class 4 to 37 in super-class 2 of TRANSFAC. Actually, most of the specific factors found in *Plasmodium *belong to three TRANSFAC super-classes:

0SC: a group of 27 TAP of the AP2 family proteins preferentially encountered in plants was recently annotated by Balaji and co-workers [25] and listed in the directory. Very recently, three factors of this family have been analysed at the molecular level [16,17].

2SC: Zinc-coordinating DNA-binding domains with 37 putative factors including diverse sets of Zinc finger proteins all implicated in interaction with nucleic acids.

3SC: Helix-turn-helix with seven members of the Myb family sub-class encompassing one to three tryptophan domains. The functional activity of two members has been studied: PfMyb1 (PF13_0088) [5,15] and PfMyb2 (PF10_0327) (Coetzer and Vaquero, unpublished).

One annotation within the 4SC beta scaffold factors and no annotation could be made for proteins of the 1SC Leucine zipper family. Hence, not all families of factors annotated so far in eukaryotes were found in the parasite.

In summary, the different members of TAPs in *P. falciparum *gave a total of 202 proteins. Our selection included the 129 TAPs from Coulson, 20 from Callebaut, 27 ApiAP2 from Balaji, 27 from PlasmoDB and our 34 new annotations. The relationship between our predictions and the three previous reports is summarized in the Venn diagram presented in additional file 2.

The genome of *Plasmodium *comprises 56 GTFs (Table 1 and Table 2, class I). Hence, the number of the putative general transcription factors, composing the enormous protein complex governing transcription initiation and interacting with the basal promoter region, appeared quite similar to that observed in other eukaryotes [41]. Since the GTFs are reasonably well conserved, it might be assumed, even though molecular functional analyses are still lacking, that they govern transcription initiation and elongation as in other eukaryotes [26,42].

As for the proteins involved in the structure of nucleosomes (histones), in the modulation of nucleosome compactness and last but not least the modifying enzymes in charge of the epigenetic alteration of TAPs, 63 CTFs proteins were identified (Table 1 and 3, class II). All sets of modifying enzymes are present in *Plasmodium*. The number of the putative CTFs resembles, as observed with GTFs, that of other unicellular eukaryotes and principally at the level of histone proteins and proteins involved in the histone code. Again, *Plasmodium *epigenetic regulation might be like that of other eukaryotes. Finally, our selection globally looks like that of Horrocks et al. [9], even though we excluded a few proteins and included several additional ones.

Only 73 STFs (Table 1 and 4, class III), known in eukaryotes to interact specifically with DNA motifs within the gene promoters and therefore contributing to the fine tuning of transcription level, were observed in *Plasmodium*. This number was quite similar to that of epigenetic TAPs (63) however, far less than the number encountered in other unicellular eukaryotes with a similar number of ORFs such as *S. cerevisiae *and *S. pombe*. In addition, the diversity of classes and sub-classes of the STF when compared to the TRANSFAC list is lower in *Plasmodium *with essentially two families of factors - 27 ApiAP2 first reported in plants [43] and a large variety of zinc finger proteins (37). This paucity of STFs, which has led members of the community to predict that gene regulation might be accomplished through a major contribution of epigenetic DNA or post transcriptional mechanisms, may be a consequence of not having looked hard enough or having an incomplete knowledge of novel original classes of proteins that might regulate transcription in *Plasmodium*. It might also result from poor annotation of proteins, when computational approaches are based on homology of amino acid sequences probably too far from those of other eukaryotes. It is noteworthy that in the case of GTFs their number increased when annotation was based on HCA program and the structure of functional domains that have been known for decades to be more conserved than amino acid sequences.

Finally, the annotation of regulatory elements in *Plasmodium falciparum *is probably very poor due to the highly A-T rich genome. However, the search for binding domains [19,44,45] within the promoters interacting with specific transcriptional factors is becoming a fashionable query. Therefore, we think that *Plasmodium *shares more components involved in mRNA synthesis machinery than previously envisaged. Nevertheless, *Plasmodium *might keep some originality, for example specific DNA-protein interactions yet to be predicted and different from those listed in TRANSFAC database since the A/T composition within the gene promoters might reach up to 90%.

### II. Molecular characterization of several annotated TAP

In contrast to the number of *in silico *annotated putative TAP very few of them have been analysed at the molecular level, essentially histones and proteins involved in epigenetic regulation, highlighting that molecular validation of the factors has to be expanded. Some representative reports dealing with molecular and functional studies of several sets of TAPs are indicated in the right column of additional file 1 and will be briefly reviewed. The function of very few GTF has been studied and most of the articles reporting such functions were published many years ago. Most publications concern the TBP [46-48] and the PIC [49].

Within the CTFs, some of the histones composing the nucleosomes have been investigated since 1992 [23,50]. Two of the HMGB factors, among the four annotated, were investigated as to their function and interaction with DNA structures [36]. Finally, a number of reports were published on various modifying enzymes implicated in the modification of the proteins involved in the overall histone code. These post-translational modifications (triggered by HATs, HDACs, SETs, Sir, etc.) play a role in gene regulation. Recently, the PfHP1 (PFL1005c) [34] was described as playing an important role in chromatin dynamics during antigenic variation and silencing of the var genes [34,39].

In contrast to the number of reports dealing with proteins involved in epigenetic regulation, there are only a few descriptions of STFs. Several unidentified proteins have been reported to bind DNA sequences [2,18] or DNA motifs [44] present in some *Plasmodium *promoters. In addition, very few STFs believed to interact with cognate DNA binding motifs were studied *per se *and validated molecularly: PfMyb1 (PF13_008) [5,15] and recently, three members of the ApiAP2 family (PF14_0633, PFF0200, PF11_0442) [16,17].

### III. Transcriptome profiles of the putative TAP during the erythrocytic development of *P. falciparum*

We analysed the transcriptome profiles of the 202 TAP retained in this study, throughout the erythrocytic development of *P. falciparum*. To this end, we used data generated in two independent high-throughput transcriptome analyses from the De Risi's and Winzeler's groups [6,7]. It is important to emphasize that there are substantial differences between the microarray designs of the two groups. In De Risi's study, 70 mer long oligonucleotides were immobilized and the transcript levels were monitored every hour during the ~48 hr of the erythrocytic cycle as a ratio of a mixed population of transcripts. The data represent the transcript expression profile underlining the relative maximal and minimal levels of expression. In Winzeler's study 25 mer oligonucleotides covering the entire *Plasmodium *genome were used. Their analysis was restricted to different stages representative of the intra-erythrocytic cycle (from ring to schizont and merozoite) as well as sporozoite and gametocyte stages. Some genes were absent from De Risi's data while present in Winzeler's data due to incompleteness of annotations at the time of array design. The latter data were proposed by the group to be more accountable for the relative expression level of every transcript and therefore complementary to the figures given by De Risi's analyses.

I. A phaseogram of the IDC transcriptome was created as indicated in [6] ordering the transcriptional profiles of all TAPs presented in De Risi's data sets. Examination of the time course of all transcripts of the four classes of TAPs showed a cascade distribution moving from maximal expression early in erythrocytic cycle to maximal expression thereafter (right part of additional file 3A and 3B). The transcripts were subdivided in seven different groups according to their temporal expression: from maximal expression during the early stages of development (group 1 and 2) to maximal expression later in the erythrocytic cycle (group 5-7). The temporal modulation of transcripts as regards to their putative functions emphasized a wide distribution within the seven sets of transcripts even though enrichment of some sub-classes was observed in particular sets. Class I transcripts and sub-classes corresponding mainly to RNA polymerase II and general cofactor subunits implicated in transcription initiation and elongation appeared more in group 2 and 3 at the onset of parasite cycle than in the subsequent groups followed by the *ccr4-not *transcripts later in the cycle. This was expected since these factors contribute to the basal transcriptional machinery. In contrast, all members of classes II and III including all sub-classes were extensively distributed throughout the erythrocytic cycle. Moreover, Winzeler's data, concerning six erythrocytic stages (left part of additional file 3), were compared to the 48 hr-De Risi's phaseogram. Even though it is not easy to compare such diverse time course experiments, after normalization of the data by the mean of the intensities of each RBC cycle, a good correlation between the two sets of data was observed strengthening the quality of the analyses.

**Figure 1:**
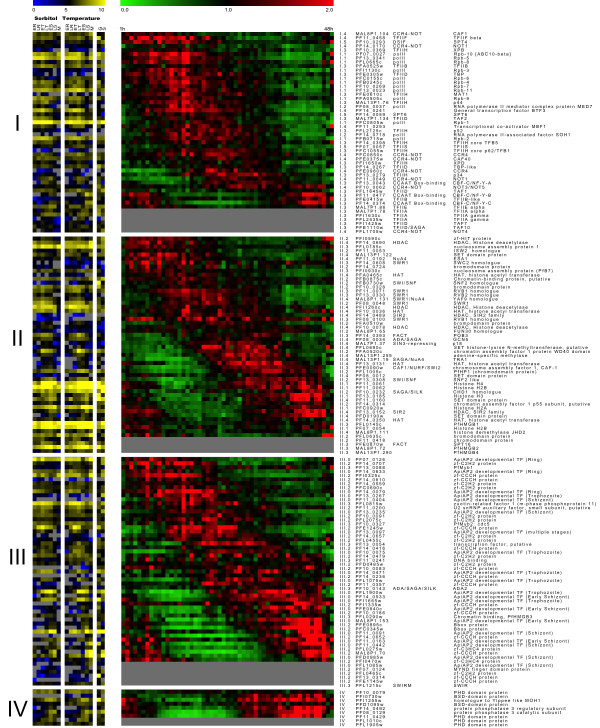
**Comparison of the De Risi's and Winzeler's TAP transcriptome data**. A phaseogram of De Risi data (centre) was created for every super class of TAPs (I to IV). Winzeler's transcriptome data from two synchronized 3D7 cultures (sorbitol and temperature) were compared gene by gene to De Risi's data (left). The colorimetric representation of De Risi's data is from green to red and Winzeler's blue-yellow (low to high) in line with the different stages of the IDC (ER and LR: early and late rings; ET and LT: early and late trophozoites; ES and LS: early and late schizonts; M: merozoites, G: gametocytes; S: sporozoites). To the right to the phaseogram, the first lane represents the classes and subclasses of TAPs, followed by accession number, and if appropriate the corresponding protein complex and finally the functional annotation.

II The phaseogram issued from De Risi's group was ordered according to every class of TAPs from I to IV and then compared to Winzeler's data that evaluate better the transcript levels, therefore leading to complementary information (Figure 1). In figure 1I, where only the GTFs (class I, left of the figure) were examined, maximal expression of transcripts encoding different RNA polymerase subunits implicated in transcription of the three classes of RNA (messenger, ribosomal and transfer RNAs, respectively, *rbp 3, 5, 8, 10*) and one transcript encoding the TBP (PFE0305w) appeared somewhat earlier in the cycle than those of RNA polymerases II subunits only contributing to messenger transcription (*rbp 1, 2, 4, 6, 7, 9, 11*). This was followed by maximal expression of transcripts encoding diverse members of the TFII subunits and CCR4-NOT along with the CAAT and TBP-like proteins. In addition, it can be seen in Winzeler's data that several RNA pol II subunits (*rbp1, 2, 5, 10, 11*) were highly expressed as well as some of the TFII subunits in contrast to others. Finally, some of these transcripts were found preferentially either in gametocytes [*tfIIa *(MAL7P1.78), *tfIId *(PF14_0267) and *tfIIf *(PF11_0458)] or in sporozoites [*tfIId *(PFL1645w) and one *ccr4-not *(PFL1705w)] when compared to the erythrocytic cycle, indicating some specificity of expression within diverse stages of parasite development.

In figure 1 II, maximal and minimal expression of transcripts encoding different CTFs and in particular several members of the different sub-classes of modifying enzymes such as HDACs, HATs and SETs proteins were distributed over the cycle, as observed for diverse members of the general factors. Moreover, some members of the modifying enzyme sub-class (HATs and HDACs) appeared to be highly expressed whereas others showed a low level of expression. As expected, high expression of every histone transcript, corresponding to the most abundant proteins encountered in eukaryote nuclei, (eight annotated in PlasmoDB) was also observed and maximal expression was evidenced in the last part of the cycle (along with the *hmgb *transcripts). It is noteworthy that two pairs of histone genes (*h3-h2a and h4-h2b -*PFF0865w-PFF0860c and PF11_0061-PF11_0062) shared one bidirectional promoter. The time course analysis of transcript expression of the *h4-h2b *genes revealed that they were probably co-regulated compared to the *h3-h2a *transcripts. In addition, in Winzeler's data, *h4-h2b *were highly expressed, as expected for histone genes throughout erythrocytic development and in gametocytes whereas *h4 *was less expressed in sporozoites than *h2b*. Information on the level of expression of *h3-h2a *transcripts is lacking in Winzeler's data. Particular attention was paid to the temporal expression of three *pfhmgb *transcripts: only *pfhmgb1 *was present on De Risi's microarray with a transcriptome profile resembling those of histone transcripts. In contrast, in Winzeler's microarrays the three *pfhmgb1*, *2 *and *4 *transcripts were present. As expected, the transcripts of both *pfhmgb1 *and *2 *were markedly expressed throughout the erythrocytic cycle with higher expression of *pfhmgb2 *in gametocytes than *pfhmgb1 *in good agreement with our previous results [36].

In figure 1 III, as expected from the previous observations, the transcripts of different members of a given sub-class of STF and in particular the 37 Zinc finger, 27 ApiAP2 and eight HTH factors were distributed all over the phaseogram. In addition, as aforementioned, some of these factors are diversely expressed. Concerning the three ApiAP2 factors [16,17] that have been molecularly analysed, the transcript of ApiAP2 ring (PF14_0633) elicited maximal expression earlier in the erythrocytic cycle than ApiAP2 schizont (PFF0200) and (PF11_0442). Maximal expression of the others was distributed throughout the phaseogram. In addition, some ApiAP2 were preferentially expressed either in sporozoïtes or gametocytes. For the HTH factors PfMyb1 and PfMyb2, the maximum of expression of *pfmyb1 *(PF13_0088) occurred earlier in the cycle than that of *pfmyb2 *(PF10_0327). The level of *pfmyb1 *expression is in good agreement with our previous report where we showed that *pfmyb1 *was poorly expressed and PfMyb1 protein was present more in the ring nuclear extracts than later in the cycle when analysed by EMSA approach [5]. The finding that various members of a family of specific transcription factors are spread over the cycle or sometimes expressed in specific stages of the erythrocytic cycle was expected. This observation underlines their functional diversity in transcriptional regulation of different sets of transcripts according to the interacting domains and their presence on the promoters of different sets of genes. In addition, it is important to determine the differential expression of some transcripts in all stages of parasite development including gametocytes and sporozoites. It is obvious that several TAP transcripts are preferentially expressed in gametocytes and others in sporozoites corresponding to differentiated and arrested stages, in contrast to proliferating stages from rings to schizonts. Finally, it is interesting to associate mRNA and protein expression. This will be discussed further when expression of the cognate proteins will be analysed and compared to transcript expression.

III. All transcripts issued from Winzeler's data were clustered by K-means method within five different clusters ranging from low (blue code colour, top) to high (yellow code, bottom) expression of transcripts (see additional file 4). This data presentation was used to highlight the highly expressed transcripts within the parasite. It appears that diverse members of several classes and sub-classes of TAP were expressed differentially throughout erythrocytic development as well as in gametocyte and sporozoite stages. In clusters 1 to 3, the level of expression was low and often a lack of data (gray code) was observed due to undetectable expression of transcripts among which members of all sub-classes of the four classes of TAPs. Additionally, some transcripts were preferentially expressed in gametocytes: two *Zn-CCCH *(PF13_0314 and PFF0095c), *SNF2 *(PFB0730w), *TBP-like *(PF14_0267) and *histone h3 *(PF13_0185). Several others, including *ApiAp2 *(PF13_0267, PF13_0235, and PFL1900w) *and Zf-c2 h2 *(PF14_0707), were more expressed in sporozoites than during the erythrocytic stages. A wide distribution of different transcript members was observed also for clusters 4 and 5, where transcript expression clearly reached a high level. Transcripts encoding histones and NAPs involved in nucleosomal and epigenetic transcriptional regulation are highly expressed as observed in all eukaryotes. Actually, Winzeler's data monitored five histone transcripts present in cluster 5 (including the tandem:*h4-h2b*, PF11_0061 and PF11_0062), one *h3 *(PFF0510w), one *h2a *(PFC0920w) and one *h2b *(PF07_ 0054). The *h3 *(PF13_0185) transcript appeared poorly expressed (cluster 3) in contrast to the others, but was highly expressed in gametocytes. In contrast to *pfmyb2 *(PF10_0327, cluster 4), the low expression of *pfmyb1 *(PF13_0088, cluster 1) is in good agreement with our previous report [5]. Finally, the *ApiAp2 *transcripts were also found in all clusters therefore indicating a wide variation of their expression levels. As for the two reported *ApiAp2 *[16], one does not even appear in cluster 1 (PFF0200c as well as PF11_0042 [17]), in contrast to the other (PF14_0633, ring associated, cluster 5) which in addition was not expressed in gametocytes but in sporozoites. Finally, some of the transcripts were more highly expressed throughout the erythrocytic cycle in contrast to others preferentially expressed either in sporozoites or in gametocytes such as several members of transcripts encoding ApiA2 and Zinc finger proteins.

In summary, the large diversity in the levels of TAP transcripts observed during the *Plasmodium *erythrocytic cycle is essential to understand the functional differentiation observed during the complex development of the parasite within the human and mosquito hosts. In the case of STFs such as the ApiAP2 family, the availability of the encoded proteins and the hierarchy of interactions between the different members (from high to low) with a variety of cognate DNA regulatory elements displayed in the gene promoters would affect the efficiency of gene expression governing the fine tuning of transcript expression throughout the development of the parasite. In addition, some transcripts are highly expressed in *Plasmodium *such as histone and *hmgb *transcripts. In this respect, again, *Plasmodium *resembles the other eukaryotes since these proteins are known to be the most highly expressed in eukaryote nuclei.

### IV. Genomic localization of TAP

**Figure 2:**
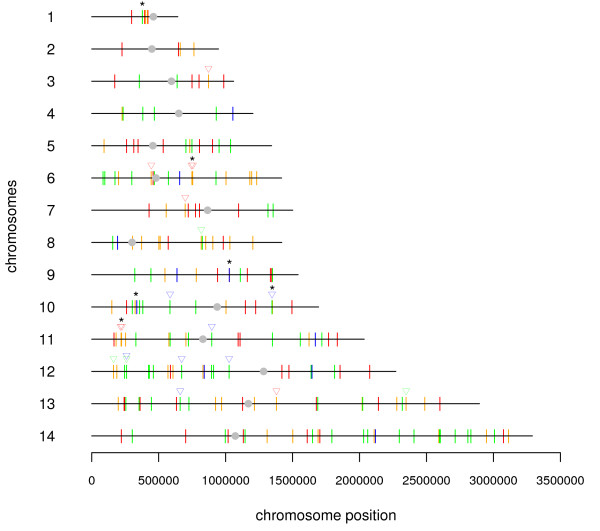
**Genomic localization of the TAP over the different chromosomes**. The chromosomes are numbered on the left and centromeres are indicated in grey. The colours of the lines correspond to the different classes of TAPs: red, general transcription factors; orange, chromatin-related factors; green, specific transcription factors and blue, TAP partners and the * indicates the six clusters. The triangles above the lines are: red for histones; blue for Myb and green for HMGB.

To determine whether genomic organization of TAP genes might govern coordinated expression of genes, the localization of transcript clusters was analysed. All 202 TAP open reading frames were mapped along the genomic sequences of the 14 chromosomes. A wide distribution of the TAP ORFs and in particular all members of the diverse TAP families was observed over the 14 chromosomes with the exception of the telomeric regions where no TAP genes were found; as expected (Figure 2). In addition, there are very few *bona fide *clusters of TAPs, only six with a low number of genes and located in different chromosomes (although in some regions several other TAPs appeared loosely clustered). In chromosome 1, a cluster of five genes composed of several GTF sub-units (RNA pol II rpb9 and TFIIB (PFA 0505c and PFA0525W), a CTF (PFA0520c) and a STF with bromo domains (PFA0510w) was found close to the centromere. Their maximal expression occurs rather early in the *Plasmodium *cycle (cluster 2 to 4 of additionnal file 3A). As for the eight histone genes (red triangle) they are present in chromosomes 3, 6, 7, 11 and 13. Two tandems of bidirectional genes *h3-h2a *and *h4-h2b *are observed in chromosome 6 and 11, respectively. Chromosome 6 also contains an additional histone *h3 *gene close to the centromere. A third putative histone *h3 *is present in chromosome 13. Therefore, the presence of three different histone *h3 *genes might be important since expression of specific members might occur at different stages of parasite development. The maximal expression of histone genes occurred fairly late in the cycle, along with parasite proliferation, with a close coordinated expression, especially for *h4-h2b*. In chromosome 9, a two gene cluster was observed encoding an HDAC and a yippee like protein, a putative zinc binding protein listed in class IV partners (PFI1260c and PFI1255w). However, they are not co-regulated. Finally, there are two additional two gene clusters in chromosome 10, fairly well co-regulated comprising the transcripts for PfMyb2 (PF10_0327) and for a bromo domain putative protein (PF10_0328) (class II and class III), the second one also comprising an HDAC PF10_0078 and PF10_0079 encompassing several PHD and AT hook domains (class II and IV).

In summary, there are only a few TAP genes organized in small genomic clusters in *Plasmodium *and only a loose co-regulation of expression was observed for these clusters as repeatedly encountered in eukaryotes. There again, *Plasmodium *behaves like other eukaryotes.

### V. Analysis of the proteome profiles and comparison with the transcriptome data

**Figure 3:**
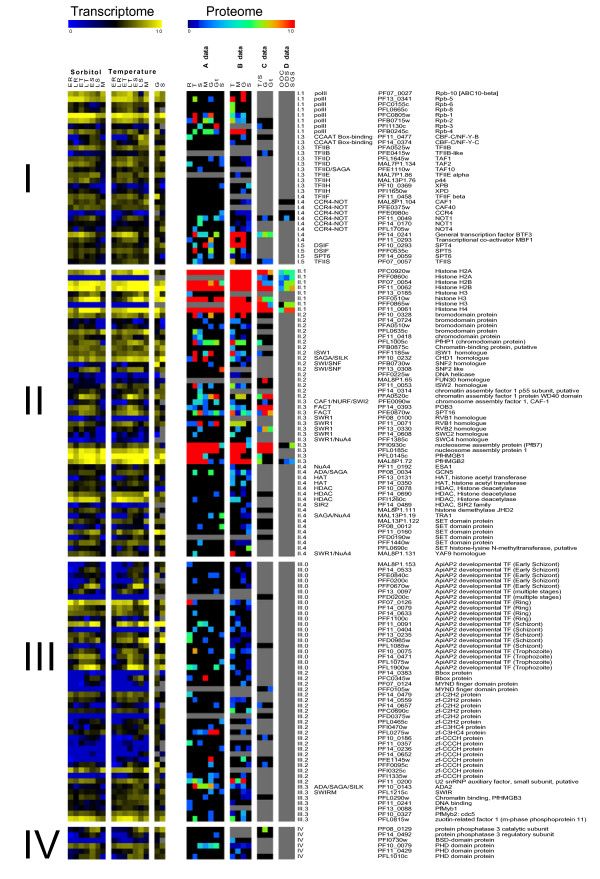
**Comparison of transcriptome and proteome data issued from Winzeler's, Florens' and Lasonder's reports**. Winzeler's transcriptome data, obtained from two different culture synchronizations (see Figure 1), was ordered according to the I-IV class of TAPs and compared to the proteome data obtained from A, B, C and D reports (from left to right) [29-32]. The colorimetric representation of the transcriptome is as in figure 1. The colorimetric representation for the proteome is black-blue for low, orange-red for high as indicated on top of figure. Grey represents absence of detection. Details for each TAP are as in the previous figures.

The proteome data are based on four different reports [29-32] that, for simplification, we will refer to in the following paragraph as A, B, C and D for Le Roch, Florens and the two Lasonder's reports. Figure 3 shows on the left, the A transcriptome data with respect to every class of TAP from I to IV and seven different parasite stages of erythrocytic development as well as gametocytes and sporozoites (indicated top of the figure) for comparison with their cognate proteins. For A and B proteome reports, the same methodology was used based on MudPIT followed by mass spectrometry. The A data reported the identification of 130 TAP of the 202 listed in the directory (Table 1) and B data 114. Most of the factors were identified by both groups, while a few of them were identified only by C. The methodology used by C and D was based on 1D gel protein fractionation of mainly the gametocyte stages and parasite stages from the mosquito followed by MS. They reported only 47 TAP including several additional proteins not detected by either group A or B. It is noteworthy that the total number of proteins determined by the A, B and C groups (139 proteins, including the six additional of C) throughout erythrocytic development represents around half the number of transcripts. It has been already stated that the number of identified proteins is markedly lower than that of transcripts most probably due to the different sensitivity of the experimental approaches. Indeed, the global analysis in *Plasmodium *described 4294 transcripts with only 2904 cognate proteins detected in the investigated erythrocytic stages [29].

In figure 3 we compared the A transcriptome profiles to the semi-quantitative evaluation of cognate proteins with a colour code from black-blue (low expression) to orange-red (high expression) and some representative examples are presented. The comparison of the A transcriptome with B and C proteome data appeared difficult to establish because only trophozoite or trophozoite/schizont stages were investigated for protein expression. Only the comparison between A transcriptome (rings, trophozoites and schizontes) and proteome data can be correctly considered. However, we extended our examination to the merozoite, gametocyte and sporozoite data.

First, some highly expressed transcripts showed high expression of their cognate proteins indicating a correlation between their levels of expression. Considering the three proteomics data, most of the histone proteins were highly expressed during the erythrocytic cycle, either as transcripts or as proteins, albeit histone *h3 *and *h2a *were not seen in the A transcriptome and proteome experiments. Tandem proteins [H4-H2B (PF11_0061-PF11_0062)] were highly expressed at each stage and to a lesser extent tandem [H3-H2A (PFF0865w-PFF0860c)]. Conversely, some discrepancy was observed between expression of transcripts and proteins in gametocytes and sporozoites, especially in the case of one H2A protein (PFC0920w). The two PfHMGB proteins are highly expressed throughout the cycle, as their cognate transcripts, with a substantial correlation observed during the erythrocytic cycle. However, in gametocytes expression of PfHMGB2 (MAL8P1.72) is high in contrast to that of PfHMGB1 (PFL0145c) in good agreement with our previous results showing a preferential expression of PfHMGB2 in gametocytes [36]. PfHMGB2 is also highly expressed in sporozoites in contrast to the PfHMGB1 counterpart. Another set of highly expressed proteins, the two NAP (PFI0930c and PFL0185c) are highly expressed in gametocytes [35] but not in sporozoites.

Second, discrepancy between levels of transcript and protein expression was observed, either high transcript levels associated to low protein levels or *vice versa*. There are several cases of TAP proteins highly expressed with low expression, if any, of their cognate transcripts NAP Pfb7 (PFI0930c) or specifically detected in some particular stages of *Plasmodium *cycle. For example, Rpb3 (PFI1130c) is expressed essentially in gametes (A data) and H2A (PFC0920w) in gametocytes and sporozoites in both A and B proteome data with almost no detection of the corresponding transcripts. This suggests a specific expression and therefore a key role of this factor in these *Plasmodium *stages. This is also true for a SPT4 (PF10_0293) and Bbox (PFC0345w) proteins highly expressed in merozoites with nearly no cognate transcript. In contrast, some mRNA, found in either class of TAP, were highly expressed with low amounts of cognate proteins. Two HDAC (PF14_0690 and PFI1260c), various SET proteins including SET (PF11_0160), one Zf-CCCH protein (PFI0325c), one PfMyb-related factor [Zuotin-related protein (PFL0815w) and PfMyb2 (PF10_0327)] were poorly expressed, even undetectable either in the B and/or C experiments. Several ApiAP2 transcripts were also highly expressed (PF07_0126 and PF11_0091) with conversely weak expression if any of the corresponding proteins. Both transcriptional and proteomic methods depend on having accurate gene models and some discrepancies could be due to the fact that gene boundaries may be incorrect for up to 20% of genes [51]. Finally, more puzzling is the poor correlation observed between the three groups of proteome data. Whereas Rbp1 protein is observed at a high level in trophozoites and gametocytes in the A data, it is poorly detected in B and C experiments indicating that the mass methodology used to investigate protein expression probably plays a significant role in these divergent results.

In summary, as could be expected, correlation between the levels of transcripts and proteins is not always observed [29,52] and this is also true for TAPs. It has been known for a long time that in eukaryotes gene expression involves regulation at the level of transcription and post-transcription, including maturation, transfer of the mRNA into the cytoplasm, as well as translational and post-translational regulation and also at the level of protein half-life. In eukaryotes, protein translation exerts an important control on gene expression from high to low levels of protein amplification depending on the structural characteristics of the mRNA, which are far from having been analysed in *Plasmodium*. It is reasonable to think that *Plasmodium *shares with other eukaryotes many features of the machinery of translation accountable for the regulation of protein synthesis. If we focus on translation regulation, some transcripts can be classified as "strong ", i.e. a low level of mRNA efficiently translated leading to a marked amplification of the cognate proteins. The reverse is also observed, "weak mRNA" expressed at high levels associated to low levels of encoded proteins. This discrepancy between transcript and protein expression can be explained by true regulation at the level of translation, as already reported for some genes and particularly during sexual differentiation [11]. However, this can also be explained by limitations of the various experimental high through put approaches and at the moment it is difficult to evaluate the contribution of true translational regulation and/or poor protein evaluation along with its quantification. Finally, a huge number of additional functional experiments have to be performed to determine the overall contribution of transcription and translation in gene regulation. That will require real motivation to undertake gene by gene analyses.

### VI. Analysis of the interactome of all TAP

In eukaryotic cells, proteins generally function as part of large protein complexes and this is true for the multifactorial complex machinery of transcription involved in the transcriptional network. In *Plasmodium*, it is reasonable to assume that the TAPs, as in other eukaryotes, are part of protein complexes. Therefore, we searched the literature for protein-protein interactions and interactome reports in *Plasmodium*. Only an extremely low number of interactome reports have been published so far based on *in silico *[53] and yeast two-hybrid (Y2H) high-throughput experiments [54] and compiled in PlasmoDB database [55]. We extracted from these reports all possible protein interactions and established a network of 2682 proteins with 8767 potential interactions (5960 for the *in silico *study and 2811 for the Y2H approach). It should be noticed that only four interactions were common to the two studies (data not shown).

**Figure 4:**
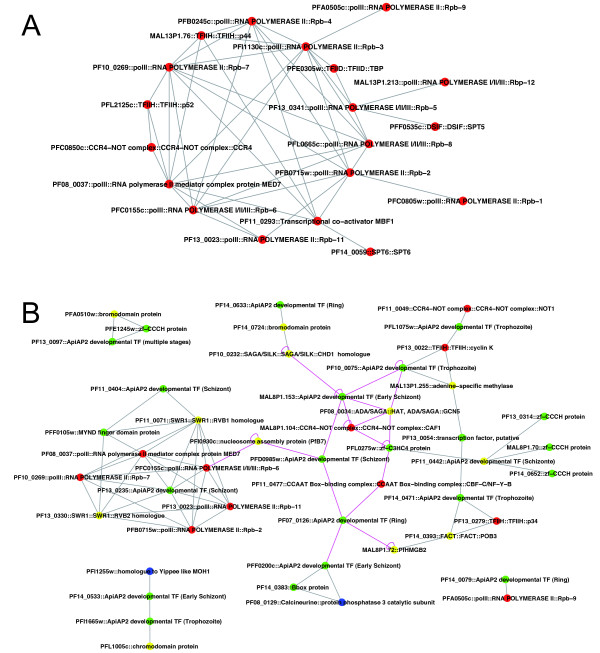
**Potential interactions observed by *in silico *and yeast two hybrid approach between the GTF and ApiAP2**. TAP candidates eliciting interactions from *in silico *[53] and Y2D data [54] were extracted from PlasmoDB and only the proteins for which an interaction was inferred are presented. The two networks were merged and graphically represented using Cytoscape 2.6 http://www.cytoscape.org. Red circle stands for general transcription factors, yellow for chromatin-related proteins, green for specific transcription factors and blue for partners. Grey lines represent the *in silico *interactions and pink lines those proposed by the two-hybrid experiment. A. Interaction network for the preinitiation complexe. B. Interaction network for the ApiAP2 TF. For the complete interaction network see Additional file 5.

Among the 202 TAPs of each class, around half were predicted to interact by *in silico *and Y2H approaches and far more interactions were detected with *in silico *method [27 of the 56 GTFs (red points), 35 of the 63 CTFs (yellow), 43 of 73 STFs (green) and three of the 10 partners (blue)]. The network of these factors, presented in figure 4 and additional file 5, was constructed as indicated in Methods, from the *in silico *(gray lines) and Y2H (pink lines) studies. The figure 4 is a blow up of interactions between the factors composing the PIC and between ApiAP2 with other TAPs. For the GTFs, the *in silico *data showed, as expected (Figure 4A), significant interactions between a number of RNA polymerases and TFII subunits composing the PIC, plus interactions with several CTFs and STFs (additional file 5), including several ApiAP2, Zf-C2H2 and PfMyb2 (PF10_0327) [53]. In contrast, no molecular interaction was detected by the Y2H approach between the PIC components, strongly highlighting that this experimental procedure missed many interactions and therefore the lack of robustness of the data. Figure 4B presents the interactions of the ApiAP2 including three ApiAP2 factors [16,17] that have been molecularly analysed: ApiAP2 ring (PF14_0633) and ApiAP2 early schizont (PFF0200, PF11_0442). Indeed, very few interactions were depicted in La Count's data (pink lines in additional file 5), for example some members of ApiAP2, Zinc finger and Myb as well as several GTFs and CTFs. The rbp6 polymerase was shown to interact with the NAP- PfB7 (PFI0930C). The best networks were centred around ADA/SAGA:GCN5 (PF10_0232) a SAGA protein [54], ADA (PF08_0034) and an ApiAP2 (MAL8P1.153). Finally, the total lack of overlap between the results based on these two approaches reflects their poor reliability.

In summary, when interactions were investigated among the 202 TAP, even for the basal machinery of *P. falciparum *which is conserved in its main components almost certainly generating the PIC as in other eukaryotes, the lack of Y2H interactions and the lack of overlap between *in silico *and molecular analyses lead to low, if any, confidence in these data. Nevertheless, an improved prediction of interactions would contribute to a new annotation and further functional validation of potential STFs that are without any doubt required, as in other eukaryotes, to govern the level of transcription. However, when interactions between the TAPs and all *Plasmodium *proteins were examined, far too many protein interactions were observed. This, together with the total absence of overlapping results, suggests the fallacy of both computational and Y2H data. Further molecular gene analyses are needed to validate the *in silico *prediction and to improve the deciphering of the transcriptional network. In addition, it is essential that the proteins composing protein complexes are co-expressed within the same stages of the *Plasmodium *development and co-localised within the cell nuclei, information that is still missing. Although a few interactions between STFs and DNA regulatory elements have been described reporting the potential role in transcriptional regulation, only the target genes of two specific transcription factor PfMyb1 (PF13_0088) and APiAP2 (PF11_0442), were demonstrated by ChIP-chip and microarray analysis [15,17].

## Conclusions

Here, we propose a directory of TAPs implicated at different levels of *Plasmodium *transcriptional machinery. Their expression, throughout the erythrocytic development of *Plasmodium falciparum*, as well as their putative interactions, has been analysed. The final goal was to reconstruct the transcriptional network dedicated to messenger RNA expression. The directory is based on all reported annotations that we compared to our own *in silico *annotation. As indicated in Table 2 to 5, we listed 202 TAPs including 34 new potential TAPs, showing a high diversity of expression (Figure 1 and Figure 3) in their transcript and protein profiles and levels throughout the erythrocytic development and also within diverse members of the same family of TAPs.

It has been described that the basal machinery of *P. falciparum *is well conserved with a similar number of components accountable for mRNA initiation and elongation of transcription when compared to other eukaryotes [41]. In addition, there is a large number of potential components implicated in the epigenetic regulation of genes such as chromatin structuring (histones) and remodelling factors (HMGB). Also, a large number of modifying enzymes of the different components of nucleosomes, such as HATs, HDACs, methylases, etc, have been predicted in *Plasmodium *and probably participate in the parasite histone code thereby governing transcription efficiency. On the other hand, around 63 proteins constitute the putative set of specific TAPs, a number lower than that observed in *S. cerevisiae *the genome of which presents a similar number of genes [42].

Most of the *in silico *identification of *Plasmodium *transcription factors has been determined *via *pairwise homology comparison with those already reported and listed in eukaryotic databases. Therefore, these algorithms miss the factors that are too distant at their amino acid levels. It is known that the structure of a protein is more conserved than its amino acid sequence and a good example was provided when Callebaut *et al*. [22] using a HCA process, complemented the annotation of GTFs with 10 new proteins not identified by Coulson *et al*. [12] leading to a number of factors similar to that encountered in higher eukaryotes. Therefore, one can assume that poor annotation of STFs accounts for the apparent low number in *Plasmodium*. Poor annotation of RE occurs also in *Plasmodium*, due probably to the high A/T content of the genome. In addition, it is conceivable that a number of RE within the promoters are specific and interact with proteins not yet described in the TRANSFAC database. Improvements in the annotation of RE and TAPs are urgently needed in *Plasmodium *considering their central role in controlling the level of gene expression.

The question as to whether the dynamic control of gene expression in *Plasmodium *is similar to that observed in other eukaryotes remains open. Also, a matter of debate is the contribution of epigenetic, transcriptional and post-transcriptional regulation in the synthesis of the proteins in charge of parasite development. Several years ago, the dogma in the *Plasmodium *research community was that the contribution of epigenetic and post-transcriptional gene regulation prevails over gene transcriptional regulation involving STFs. Nevertheless, each stage of erythrocytic development requires a highly coordinated, time-dependant mechanism to control the expression of mRNA of distinct sets of genes as determined by various transcriptome and proteome reports. Recently, in addition to the steady state transcript analysis a nuclear run on study was undertaken throughout erythrocytic cycle that showed that transcriptional and post transcriptional regulation including the transcript stability [56] participate in the regulation of gene expression [44,57].

At the moment it is impossible to attest the supposedly low contribution of STFs in *Plasmodium *since this concept was based on a poor annotation of STFs, as well as of their DNA motif counterparts. It should be kept in mind that around 60% of the *Plasmodium *genome contains orphan genes. Moreover, in addition to the STFs not yet annotated but corresponding to factors listed in the TRANSFAC databank, it is reasonable to imagine that novel REs might exist within the parasite promoters probably interacting with as yet uncharacterized STFs. Lately, several reports have proposed annotations of DNA regulatory elements [44,45]. This regain of interest for REs prediction might lead the way to the description of additional specific transcription factors, since both *cis *and *trans *regulatory elements *via *their interaction are engaged in transcription regulation.

The survey of transcriptome and proteome studies points out that, for diverse families of TAPs among which the sub-units of PIC, APiAP2, zinc fingers and Myb, levels of expression of different members vary markedly throughout the erythrocytic cycle and within sporozoites and gametocytes. Some members within TAP families are more expressed in sporozoites and/or gametocytes whereas others are more expressed at given times of the erythrocytic cycle. The large diversity of TAP expression during the *Plasmodium *biological cycle is essential to understand the functional complexity of gene expression observed during parasite development within the human and mosquito hosts.

Finally, the level of transcription is governed by the binding efficiency of each factor with their cognate DNA binding motifs, even though epigenetic and post-transcriptional regulations also contribute to gene expression. The diversity of interactions between a given member of a particular STF family and a given DNA binding domain of a particular DNA motif family, is based on the variation of amino acid and DNA sequences including the flanking sequences of each binding site [58]. The modulation of expression of the targeted transcripts is the result of the combination of these different interactions. In this context a very promising anti-plasmodial strategy would be to inactivate one or more key sets of TFs with drugs. This would compromise gene regulation and hence the function of numerous downstream genes and vital biological processes, including the development of transmission stages. Since several genes would be affected, this type of approach would make it much more difficult for the parasite to develop resistance to the drugs.

## Methods

### *Plasmodium *TAPs identification procedure

We first established a list of Pfam HMM profiles by combining all Pfam HMM profiles (using HMMER 2.3.2) described in the literature [21,22,25] for the *P. falciparum *TAPs and adding Pfam HMM profiles of 109 HMM corresponding to TAP domains found in eukaryotic and prokaryotic organisms (see the list in additional file 6). Briefly, Coulson *et al*. [12] used two strategies to identify the TAPs of *P. falciparum*: 51 HMM profiles from TRANSFAC and a sequence similarity clustering method were used to identify 156 TAPs including 71 STFs. Callebaut *et al*. [22] used a two dimensional HCA to identify 10 additional TFII subunits when compared to Coulson annotation. Balaji *et al*. [25] reported the first STF family of the apicomplexan (ApiAP2) based on their description on the Pfam database http://pfam.sanger.ac.uk/. Indeed, a special AP2 HMM profile was constructed using an alignment of the ApiAP2 of *Plasmodium *proteins. Then a list of 109 Pfam HMM profiles was used to scan the whole genome of *P. falciparum *with HMMER 2.3.2 for protein annotation.

Functional annotation was assessed by 1) comparing the domain organization of each protein to the annotations of proteins showing the same domain organization in the Pfam database and 2) searching orthologs in model organisms (*S. cerevisiae*, *S. pombe, A. thaliana*, *M. musculus*, *H. sapiens)*. When the TAP candidates were annotated as belonging to a protein complex in model organisms, we searched for all *P. falciparum *orthologs of these complexes and subunits that were missed using the HMM strategy and they were added to the final list of TAPs. Finally, the 202 TAPs retained were classified into four functional classes and several sub-classes. In addition, for class 3 of specific transcription factors, TRANSFAC database classification was employed (see Table 1 to 5 and additional file 1).

### Data filtering

Transcriptomic data form Le Roch *et al*. [7] were filtered as follows: for each gene at least one intensity over 10 in the 16 experiments (this was the cutoff used in the original paper) and a probe set with at least five probes (to take into account very small ORFs such as those coding for histones or HGMGB for which only a few probes can be designed). The resulting set of genes was used to filter the trancriptomic data of Bozdech *et al*. [6] based on expression level as well as the proteomic data [29-32].

### Representation mode for transcriptome and proteome as well as for interactome data

The transcriptomic data taken from the literature [6,7] were evaluated by using TIGR Mev 4.2. Temporal ordering of erythrocytic stages of transcriptomic data was performed using the phaseogram of Bozdech *et al*. K-means Clustering was performed with TIGR Mev 4.2 using five classes and euclidian distance of genes for which there existed data in at least 12 experiments out of 16. Graphical representation of proteomic data [29-32] realized using TIGR Mev 4.2 and the genomic localization of TAPs genes was established with R using PlasmoDB 5.4 genes coordinates. TAPs showing interactions in Y2H [54] and in *in silico *study [53] with a likelihood >10 were extracted from PlasmoDB and the two networks were merged and graphically represented using Cytoscape 2.6 http://www.cytoscape.org.

## Abbreviations

CTF: Chromatin-related Transcription Factor; GTF: General Transcription Factor; HAT: Histone Acetyl Transferase; HCA: Hydrophobic Cluster Analysis; HDAC: Histone DeACetyl transferase; HMM: Hidden Markov Model; HMGB: High Mobility Group box B; HTH: Helix Turn Helix (TRANSFAC super class); mRNA: messenger RNA; MS: Mass Spectrometry; NAP: Nucleosome Assembly Protein; ORF: Open Reading Frame; PIC: Pre-Initiation Complex; RE: Regulatory Element; STF: Specific Transcription Factor; TAP: Transcription-Associated Protein; TBP: TATA binding protein; TF: Treanscription Factor; Y2H: Yeast two-Hybrid.

## Authors' contributions

EB performed all bioinformatics analyses. CV and EB conceived the study, analyzed the results and wrote the manuscript. Both authors read and approved the final manuscript.

## Supplementary Material

Additional file 1 Detailed directory of *Plasmodium *AP with a non exhaustive list of molecular reports. Click here for file

Additional file 2 **Venn diagram of TAP data taken from Coulson, Callebaut and Balaji reports and compared to our data**. Red circle represents the 202 TAP included in Table 1 to 5 and additional file 1 comprising the 104 TAP from Coulson, 10 from Callebaut, 27 ApiAP2 from Balaji, 27 from PlasmoDB and our 34 new annotations. Green circle represents the 129 annotated TAP from Coulson. Blue circle represents the 20 general TAP from Callebaut including the 10 already predicted by Coulson. Click here for file

Additional file 3 **Overview of the *Plasmodium *IDC TAP transcriptome**. A. A phaseogram of the IDC transcriptome was created as indicated in [6] by ordering the transcriptional profiles of all TAP within the 202 present in DeRisi's data and in the normalized Winzeler's data. Furthermore right to the phaseogram: first lane stands for class and subclass of TAP, followed by accession number, and if appropriate the corresponding protein complex and finally the functional annotation. This phaseogram was subdivided (from high to low) in seven sets of genes as indicated in the left of the figure. The green-red (low to high) representation of gene expression ratio is specified top of the figure. B. The hours of maximal expression reached throughout the IDC by every class of TAP are indicated: red: I. general transcription; green: II chromatin-related; blue: specific transcription factors and orange: IV TAP partners. Click here for file

Additional file 4 **Clustering of Winzeler's data according to the deduced level of transcripts potential**. The data using TIGR Mev 4.2. software were grouped in five clusters (left) from low to high expression levels. Colorimetric representation and the different stages of the IDC are as in figure 1 (top of the figure). Click here for file

Additional file 5 **Potential interactions observed within the 202 TAP by *in silico *and yeast two hybrid approach**. TAP candidates eliciting interactions from *in silico *[53] and Y2D data [54] were extracted from PlasmoDB and only the proteins inferring an interaction are presented. The two networks were merged and graphically represented using Cytoscape 2.6 http://www.cytoscape.org. Red circle stands for general transcription factors, yellow for chromatin-related proteins, green for specific transcription factors and blue for partners. Grey lines represent the *in silico *and pink lines defined interactions proposed by the two-hybrid experiment. Click here for file

Additional file 6 List of 109 Pfam HMM profiles used for annotation of the 202 TAP. Click here for file
