# Supplementary figures and images for: In silico and biological survey of transcription-associated proteins implicated in the transcriptional machinery during the erythrocytic development of Plasmodium falciparum
Source: BMC Genomics. 2010 Jan 15;11:34. doi: 10.1186/1471-2164-11-34 (PMC2821373; doi:10.1186/1471-2164-11-34)

**Bischoff  
et al. (202)**

**Balaji  
et al. (27)**

**PlasmoDB**

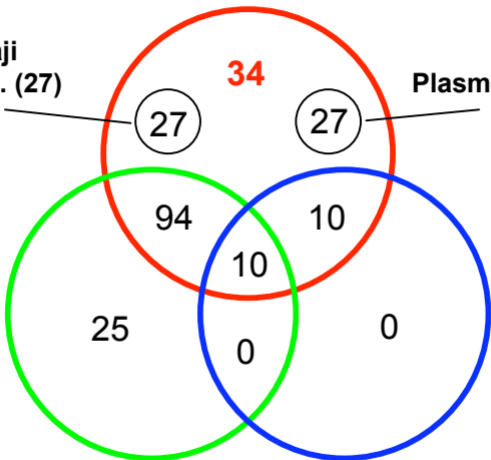

**Coulson  
et al. (129)**

**Callebaut  
et al. (20)**

Supplement: Additional file 2 — Venn diagram of TAP data taken from Coulson, Callebaut and Balaji reports and compared to our data. Red circle represents the 202 TAP included in Table 1 to 5 and additional file 1 comprising the 104 TAP from Coulson, 10 from Callebaut, 27 ApiAP2 from Balaji, 27 from PlasmoDB and our 34 new annotations. Green circle represents the 129 annotated TAP from Coulson. Blue circle represents the 20 general TAP from Callebaut including the 10 already predicted by Coulson. [file 1471-2164-11-34-S2.PDF]

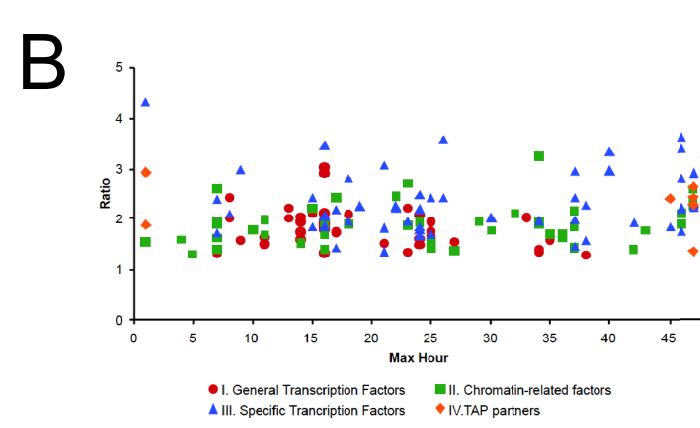

Supplement: Additional file 3 — Overview of the Plasmodium IDC TAP transcriptome. A. A phaseogram of the IDC transcriptome was created as indicated in [6] by ordering the transcriptional profiles of all TAP within the 202 present in DeRisi's data and in the normalized Winzeler's data. Furthermore right to the phaseogram: first lane stands for class and subclass of TAP, followed by accession number, and if appropriate the corresponding protein complex and finally the functional annotation. This phaseogram was subdivided (from high to low) in seven sets of genes as indicated in the left of the figure. The green-red (low to high) representation of gene expression ratio is specified top of the figure. B. The hours of maximal expression reached throughout the IDC by every class of TAP are indicated: red: I. general transcription; green: II chromatin-related; blue: specific transcription factors and orange: IV TAP partners. [file 1471-2164-11-34-S3.PDF]

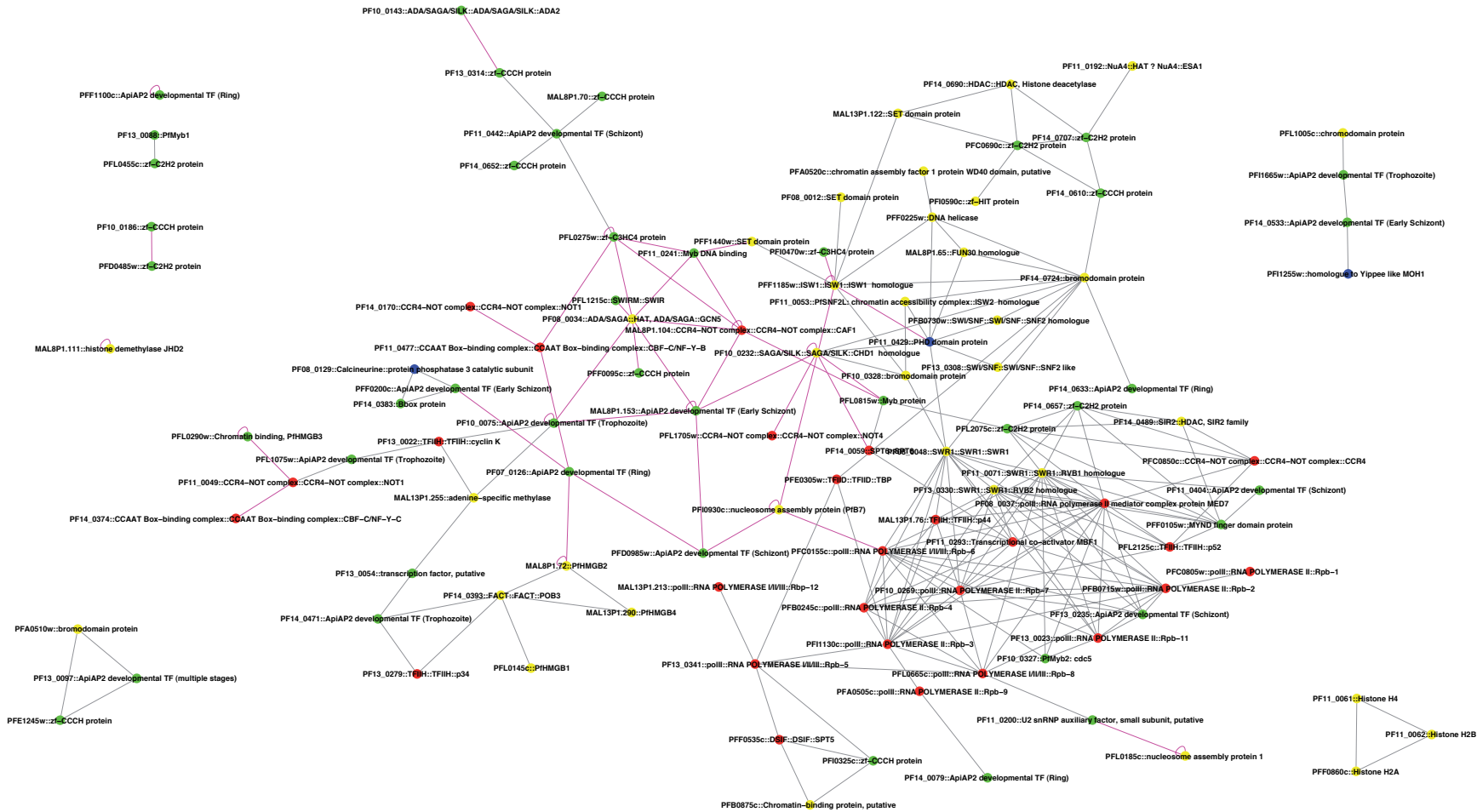

Supplement: Additional file 5 — Potential interactions observed within the 202 TAP by in silico and yeast two hybrid approach. TAP candidates eliciting interactions from in silico [53] and Y2D data [54] were extracted from PlasmoDB and only the proteins inferring an interaction are presented. The two networks were merged and graphically represented using Cytoscape 2.6 http://www.cytoscape.org. Red circle stands for general transcription factors, yellow for chromatin-related proteins, green for specific transcription factors and blue for partners. Grey lines represent the in silico and pink lines defined interactions proposed by the two-hybrid experiment. [file 1471-2164-11-34-S5.PDF]
