# Supplementary material for: In silico and biological survey of transcription-associated proteins implicated in the transcriptional machinery during the erythrocytic development of Plasmodium falciparum
Source: BMC Genomics. 2010 Jan 15;11:34. doi: 10.1186/1471-2164-11-34 (PMC2821373; doi:10.1186/1471-2164-11-34)
Supplement: Additional file 4 — Clustering of Winzeler's data according to the deduced level of transcripts potential. The data using TIGR Mev 4.2. software were grouped in five clusters (left) from low to high expression levels. Colorimetric representation and the different stages of the IDC are as in figure 1 (top of the figure). [file 1471-2164-11-34-S4.PDF]

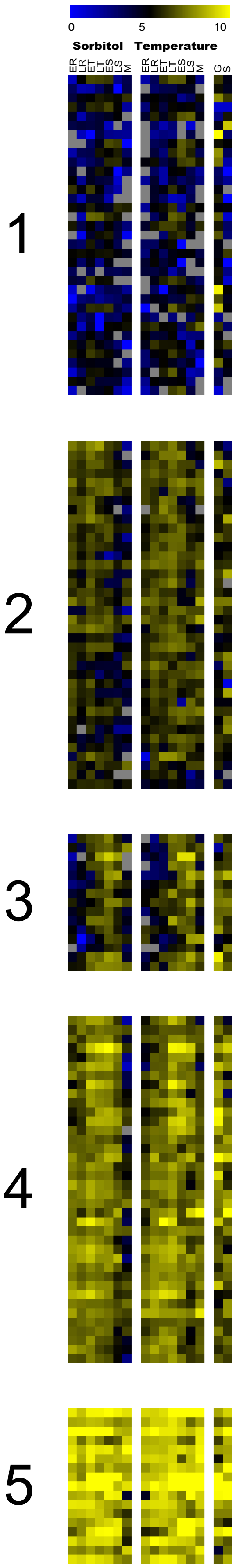

II.4	MAL13P1.255		adenine-specific methylase
II.4	PF08_0012		SET domain protein
I.4	PF10_0062	CCR4-NOT complex	NOT3/NOT5
II.4	PF10_0078	HDAC	HDAC, Histone deacetylase
III.3	PF10_0143	ADA/SAGA/SILK	ADA2
III.0	PF13_0267		ApiAP2 developmental TF (Trophozoite)
III.2	PF13_0314		zf-CCCH protein
II.4	PFD0190w		SET domain protein
III.0	PFE0840c		ApiAP2 developmental TF (Early Schizont)
III.2	PFE0895c		Bbox protein
II.4	MAL7P1.37	SIN3-repressing	p18
II.2	MAL8P1.65		FUN30 homologue
I.2	PF08_0037	polII	RNA polymerase II mediator complex protein MED7
I.3	PF10_0369	TFIIH	XPB
II.4	PF11_0192	NuA4	ESA1
I.3	PF13_0043	CCAAT Box-binding	CBF-C/NF-Y-A
III.3	PF13_0088		PfMyb1
II.4	PF13_0131	HAT	HAT, histone acetyl transferase
I.3	PF13_0279	TFIIH	p34
I.4	PF14_0170	CCR4-NOT complex	NOT1
II.2	PF14_0724		bromodomain protein
II.4	PFA0465c	HAT	HAT, histone acetyl transferase
II.2	PFA0510w		bromodomain protein
II.2	PFB0730w	SWI/SNF	SNF2 homologue
I.3	PFE0415w	TFIIB	TFIIB-like
III.2	PFF0095c		zf-CCCH protein
III.0	PFF1100c		ApiAP2 developmental TF (Ring)
III.2	PFI0470w		zf-C3HC4 protein
I.1	PFI1130c	polII	Rpb-3
I.3	PFI1425w	TFIID	TAF7
I.3	PFI1650w	TFIIH	XPD
III.2	PFL0455c		zf-C2H2 protein
II.4	PFL0690c		SET histone-lysine N-methyltransferase, putative
I.3	PFL2125c	TFIIH	p52
I.3	PFL2435w	TFIIA	TFIIA gamma

I.3	MAL13P1.76	TFIIH	p44
I.4	PF11_0293		Transcriptional co-activator MBF1
I.3	PF14_0267	TFIID	TBP-like
III.2	PFC0690c		zf-C2H2 protein
II.3	PFI0930c		nucleosome assembly protein (PfB7)
II.4	MAL8P1.111		histone demethylase JHD2
II.2	PF11_0418		chromodomain protein
III.0	PF13_0097		ApiAP2 developmental TF (multiple stages)
III.0	PF13_0235		ApiAP2 developmental TF (Schizont)
II.4	PF14_0350	HAT	HAT, histone acetyl transferase
III.2	PF14_0416		zf-CCCH protein
III.2	PF14_0559		zf-C2H2 protein
I.1	PFC0155c	polII	Rpb-6
I.5	PFF0535c	DSIF	SPT5
II.2	PFI0590c		zf-HIT protein
I.1	PFL0665c	polII	Rpb-8
IV	PFL1905w		PHD domain protein
II.4	MAL13P1.122		SET domain protein
II.3	MAL13P1.290		PfHMGB4
I.4	MAL8P1.104	CCR4-NOT complex	CAF1
II.4	MAL8P1.131	SWR1/NuA4	YAF9 homologue
I.5	PF07_0057	TFIIS	TFIIS
II.4	PF08_0034	ADA/SAGA	GCN5
I.5	PF10_0293	DSIF	SPT4
II.2	PF11_0053		ISW2 homologue
III.3	PF11_0241		DNA binding
I.5	PF14_0059	SPT6	SPT6
III.2	PF14_0707		zf-C2H2 protein
III.2	PFD0485w		zf-C2H2 protein
III.3	PFL0290w		Chromatin binding, PfHMGB3
III.3	PFL1215c	SWIRM	SWIR
I.4	PFL1705w	CCR4-NOT complex	NOT4
IV	PFD1095w		BSD-domain protein
I.3	PFE1110w	TFIID/SAGA	TAF10
III.0	PF10_0075		ApiAP2 developmental TF (Trophozoite)
III.2	PFL0275w		zf-C3HC4 protein
II.3	PF13_0330	SWR1	RVB2 homologue
III.2	PFL2075c		zf-C2H2 protein

I.4	PF11_0049	CCR4-NOT complex	NOT1
III.0	PF11_0163		ApiAP2 developmental TF (Early Schizont)
III.0	PFF0670w		ApiAP2 developmental TF (Early Schizont)
IV	PFL1010c		PHD domain protein
IV	PF08_0129		protein phosphatase 3 catalytic subunit
IV	PF11_0429		PHD domain protein
III.3	PF13_0054		transcription factor, putative
II.4	PF13_0152	SIR2	HDAC, SIR2 family
II.2	PF14_0314		chromatin assembly factor 1 p55 subunit, putative
III.0	PFL1075w		ApiAP2 developmental TF (Trophozoite)
I.3	MAL7P1.78	TFIIA	TFIIA alpha
I.3	PF14_0374	CCAAT Box-binding	CBF-C/NF-Y-C
III.2	PFL0465c		zf-C2H2 protein
II.1	PF13_0185		Histone H3
I.3	PFI1630c	TFIIA	TFIIA gamma

II.3	PF14_0393	FACT	POB3
I.4	PFE0375w	CCR4-NOT complex	CAF40
II.4	PFF1440w		SET domain protein
III.0	PFL1900w		ApiAP2 developmental TF (Trophozoite)
II.2	PF13_0308	SWI/SNF	SNF2 like
III.0	PF14_0471		ApiAP2 developmental TF (Trophozoite)
III.2	PF14_0479		zf-C2H2 protein
II.2	PFA0520c		chromatin assembly factor 1 protein WD40 domain, putative
I.1	PFB0245c	polII	Rpb-4
II.3	PFE0870w	FACT	SPT16
IV	PFI1255w		homologue to Yippee like MOH1
III.0	PFI1665w		ApiAP2 developmental TF (Trophozoite)
II.2	PFL1005c		PfHP1 (chromodomain protein)
I.3	PFL1645w	TFIID	TAF1
I.3	MAL7P1.86	TFIIE	TFIIE alpha
II.4	PF11_0160		SET domain protein
III.2	PF11_0200		U2 snRNP auxiliary factor, small subunit, putative
I.4	PF11_0458	TFIIF	TFIIF beta
I.1	PFA0505c	polII	Rpb-9
I.3	PFA0525w	TFIIB	TFIIB
II.2	PFB0875c		Chromatin-binding protein, putative
III.0	PFD0985w		ApiAP2 developmental TF (Schizont)
III.2	PFE1245w		zf-CCCH protein
II.2	PFF1185w	ISW1	ISW1 homologue
I.3	MAL7P1.134	TFIID	TAF2
II.2	PF08_0048	SWR1	SWR1
II.4	PF10_0036	HAT	HAT, histone acetyl transferase
IV	PF10_0079		PHD domain protein
III.3	PF10_0327		PfMyb2: cdc5
II.2	PF10_0328		bromodomain protein
I.4	PF14_0241		General transcription factor BTF3
III.0	PF14_0633		ApiAP2 developmental TF (Ring)
II.4	PF14_0690	HDAC	HDAC, Histone deacetylase
III.2	PF10_0091		zf-C2H2 protein
I.1	PF10_0269	polII	Rpb-7
II.3	PF11_0071	SWR1	RVB1 homologue
III.0	PF14_0079		ApiAP2 developmental TF (Ring)
I.4	PFC0850c	CCR4-NOT complex	CCR4

II.1	PF07_0054		Histone H2B
I.1	PF13_0023	polII	Rpb-11
I.1	PFC0805w	polII	Rpb-1
III.2	PFI0325c		zf-CCCH protein
II.3	PFL0185c		nucleosome assembly protein 1
III.0	PF07_0126		ApiAP2 developmental TF (Ring)
II.2	PF10_0232	SAGA/SILK	CHD1 homologue
II.1	PF11_0061		Histone H4
II.1	PF11_0062		Histone H2B
III.0	PF11_0091		ApiAP2 developmental TF (Schizont)
I.1	PF13_0341	polII	Rpb-5
I.1	PFB0715w	polII	Rpb-2
II.1	PFC0920w		Histone H2A
II.1	PFF0510w		histone H3
II.4	PFI1260c	HDAC	HDAC, Histone deacetylase
II.2	PFL0635c		bromodomain protein
III.3	PFL0815w		zuotin-related factor 1 (m-phase phosphoprotein 11)
